# Phytochemical Profiling and Assessment of Anticancer Activity of *Leptocarpha rivularis* Extracts Obtained from In Vitro Cultures

**DOI:** 10.3390/plants11040546

**Published:** 2022-02-18

**Authors:** Julia Rubio, Guisselle Arias, Christian Robles-Kelly, Evelyn Silva-Moreno, Luis Espinoza, Héctor Carrasco, Andrés F. Olea

**Affiliations:** 1 Instituto de Ciencias Biomédicas, Universidad Autónoma de Chile, Santiago 8910339, Chile; guisselle.arias.b@gmail.com (G.A.); christian.roblesk@gmail.com (C.R.-K.); 2 Instituto de Investigación Agropecuarias, INIA–La Platina, Avda. Santa Rosa, Santiago 11610, Chile; evelyn.silva@inia.cl; 3 Departamento de Química, Universidad Técnica Federico Santa María, Avenida España 1680, Valparaíso 2340000, Chile; luis.espinozac@usm.cl; 4 Grupo Química y Bioquímica Aplicada en Biotecnología, Instituto de Ciencias Químicas Aplicadas, Facultad de Ingeniería, Universidad Autónoma de Chile, Llano Subercaseaux 2801, San Miguel, Santiago 8910339, Chile; hector.carrasco@uautonoma.cl

**Keywords:** micropropagation, callogenesis, phytochemistry, gene expression, cell viability

## Abstract

Plant cell culture is a source of plant material from which bioactive metabolites can be extracted. In this work, the in vitro propagation of *Leptocarpha rivularis*, an endemic Chilean shrub with anticancer activity, is described. Different media were tested and optimized for the introduction, propagation, and rooting steps of the micropropagation process. At the end of this process, 83% of plants were successfully acclimatized under greenhouse conditions. Callus induction from the internodal stem segment was performed using various combinations of phytohormones. Green-colored, friable, and non-organogenic callus was generated with a callus induction index higher than 90%. The chemical composition of extracts and callus, obtained from clonal plants, was assessed and the results indicate that the phytochemical profiles of extracts from micropropagated plants are like those found for plants collected from natural habitats, leptocarpine (LTC) being the major component. However, no LTC was detected in callus extract. HeLa and CoN cells, treated with LTC or extract of micropropagated plants, exhibit important diminution on cell viability and a drastic decrease in gene expression of *IL-6* and *mmp2*, genes associated with carcinogenic activity. These effects are more important in cancer cells than in normal cells. Thus, micropropagated *L. rivularis* could be developed as a potential source of efficient antiproliferative agents.

## 1. Introduction

*Leptocarpha rivularis* DC, the common name of which is “Palo negro”, is an endemic, monotypic and dioecious species that belongs to the *Asteraceae* family. It grows mainly in the southern part of Chile (between VII and X regions) [1], and it has been known and used for centuries as folk medicine by the Mapuche people [2]. Previous phytochemical studies of *L. rivularis* have shown that extracts obtained from leaves, bark, and flowers exhibit antioxidant, hypoglycemic, and anticancer effects [3,4,5,6,7]. These biological activities have been attributed to the presence of sesquiterpene lactones, i.e., leptocarpine (LTC) as the major component and small amounts of rivularin and the 17,18-dihydroleptocarpin derivative [8,9,10,11]. It has also been described that LTC presents important cytotoxic effects on several cancer cell lines by inducing apoptosis [10], and inhibitory activity on specific growth factors in osteosarcoma [11].

Thus, the demonstrated anti-cancer properties of *L. rivularis* have prompted a huge increase in its consumption as alternative medicine, and therefore a high demand for harvesting it from its natural ecological niche has been produced. Even though thus far there is no information to suggest overexploitation, some attempts at the vegetative propagation of *L. rivularis* have been reported [12], and in vitro clonal micropropagation has also recently been described as an interesting alternative to grow it under controlled laboratory conditions [13].

Considering that the extraction and isolation of bioactive metabolites are afforded with low yields and that the relative concentrations of active compounds are mainly determined by the geographical location where plant material is collected, it becomes crucial to have a constant and permanent supply of plant material [14,15]. Thus, with this aim we used plant cell culture, a well-known platform that has been used to produce bioactive natural products [16,17,18], to develop a successful in vitro propagation protocol. Briefly, clonal explants were produced from in vitro culture of *L. rivularis* tissue. Then, a culture media was formulated for clonal propagation and plant material generated exclusively from in vitro culture was obtained. In addition, callus formation (callogenesis) of *L. rivularis* was induced from this in vitro-produced plant material. Callus is a mass of undifferentiated cells without organization, which develops naturally in a plant because of a wound, and can stop plant development, and differentiate or multiply themselves indefinitely [19,20]. As callus do not photosynthesize, they lack lignified structures, which facilitates the extraction and identification of metabolites [21,22,23]. Therefore, micropropagation of *L. rivularis*, is intended to avoid the massive pruning and obtention of LTC compound from plant material generated exclusively from in vitro culture. The phytochemical profiles and biological activities of extracts obtained from callus and clonal explants were compared with extracts of plants collected from their natural ecological niche.

## 2. Results and Discussion

### 2.1. In Vitro Micropropagation of Leptocarpha Rivularis

The in vitro propagation platform for the micropropagation of *L. rivularis* involves three different stages, namely, introduction (I), propagation (P) and rooting (R). Plant cuttings, having at least two axillary buds each, were used for the in vitro culture introduction of plant material. These cuttings were cultured by 20–30 days in different semi-solid culture medium (Murashige and Skoog, MS) supplemented with 3% (*w/v*) of sucrose, ascorbic acid (0.05 g/L), charcoal (1 g/L), and different hormone combinations (see Table 1).

**Figure 1:**
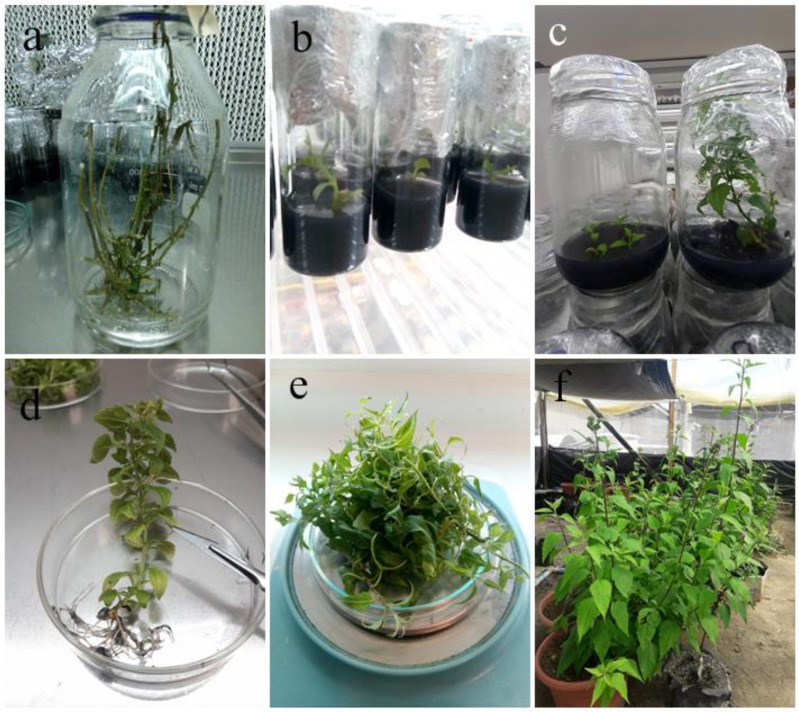
In vitro clonal micro propagation platform of *Leptocarpha rivularis*. (**a**) Stick with dormant shoot sterilization; (**b**) in vitro culture establishment; (**c**) propagation protocol for in vitro explants; (**d**) complete “adult” plant generated by in vitro culture. Uses for this material will be (**e**) semipolar extraction or (**f**) explant acclimation under greenhouse conditions.

After two weeks (I1), lateral buds sprouted, and the development of leaf blade was observed. The formulation of this medium was adjusted for the propagation stage (Table 1). In this step, explants elongated their stems, allowing micropropagation due to the abundant number of lateral shoots that they generated, considerably increasing their plant mass. A growth index of 2.0 ± 0.5 and 3.5 ± 0.5 shoots per explants was observed for introduction and propagation, respectively. Explants in P3 medium rooted spontaneously when cultured over 60 days. For rooting, explants were transferred to R1 medium for 30 days. In both mediums, 75% of the explants generated a fascicular root, which allowed obtaining 83% of successfully acclimatized plants under greenhouse conditions (Table 1, Figure 1). The explant survival rate of in vitro culture was 89.6 ± 2.7 and 95.0 ± 5.0 for propagation and rooting, respectively. These values were measured 30 days after disinfection and the beginning of spontaneous rooting, and denote the means of three independent assays with *n* = 130.

After two months of subculture, the introduced plant material of *L. rivulari*s remained free from exogenous contamination. Thus, half of the obtained explants were kept for propagation, whereas the other half was used for the generation of callus from leaves and internodal segments. The medium was renewed every 45 days, obtaining an average multiplication rate of *n* = 3. The development of protocols for the implementation of clonal micropropagation platforms in semisolid medium, using media formulated in this work, allowed obtaining *L. rivularis* explants that completed the growth and development processes in a normal way, successfully acclimatizing under greenhouse conditions and developing fertile reproductive structures. It is worth mentioning that the main difference between our results and the protocol developed by Dorta et al. [13] resides in the composition of the culture medium used in different steps of the in vitro clonal micropropagation platform of *Leptocarpha rivularis*. For example, here, mixtures of hormones (BAP and IBA) and MS (P3 and R1) were used as medium for both propagation and rooting. On the other hand, Dorta et al. used MSG/IBA medium for propagation and a free hormone MSG medium for rooting. In both cases, rooting was induced with yields over 70%. Overall, the percentage of successfully developed plants growing under greenhouse conditions is in the range of 83 to 90% for both protocols.

### 2.2. Callus Induction

Callus induction from the internodal stem segment of in vitro-generated *L. rivularis* was explored. As there is no previous information, different hormone combinations, proposed for species of the *Asteraceae* family [21,22], were tested until successful callus induction from the internodal stem segment was performed. Callus induction index (CIF) and other characteristics such as color, texture and differentiation potential were used to determine the callus formation response (Table 2).

The data in Table 2 indicate that efficient induced callogenesis is obtained in C7 and C8 mediums, i.e., having a CIF higher than 90%. These CIF values are 2–3 times larger than those previously reported for similar systems [21,24]. As result of this process, a green-colored, friable, and non-organogenic callus was generated in both medium. This is an interesting result, because it has been proposed that the development stage and tissue differentiation are as important as hormone combination to determine callus generation feasibility [26,27,28,29]. Thus, reprogramming the cell cycle in highly specialized adult cells should be a very complex process, which means that callus generation from leaves of *L. rivularis* is quite a difficult task. A plausible explanation for this high efficiency in callus induction is the use of cytokines instead of auxins, unlike other species belonging to the *Asteraceae* family [28].

**Figure 2:**
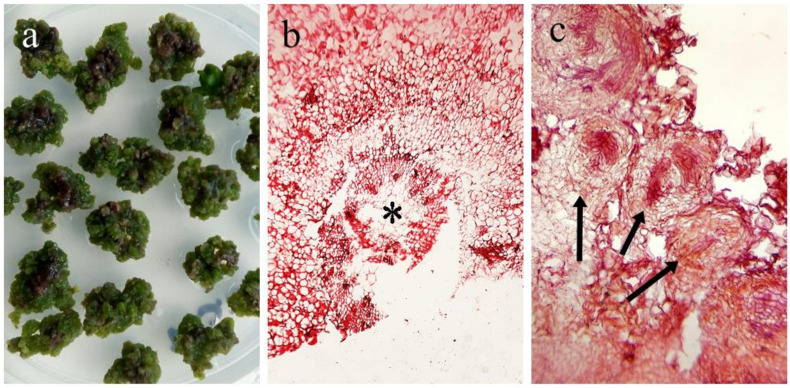
Histological analysis of *L. rivularis* callus induced under in vitro culture conditions. (**a**) Callus structures; (**b**) cells at the contact surface 21 days after callus induction, * shows an initial internodal section and following dedifferentiation; (**c**) arrows indicate meristemoids formation.

Histological analyses of these calluses are shown in Figure 2.

The photography in Figure 2c confirms the generation of undifferentiated cells grouped in round-shaped structures known as meristemoids. These are groups of adult cells that, under hormone stimuli, de-differentiate into mesenchymal cells. This group of cells are self-renewing, adopt a spiral shape and generate a large cell and smaller meristemoid that maintains its self-renewal activity, which ultimately leads to callus structure formation. Calluses doubled in mass every 60 days and were kept in culture for up to six months. Subsequently, an oxidative process began, which stopped cell multiplication and finally led to tissue death.

### 2.3. Phytochemical Analysis of Extracts

**Figure 3:**
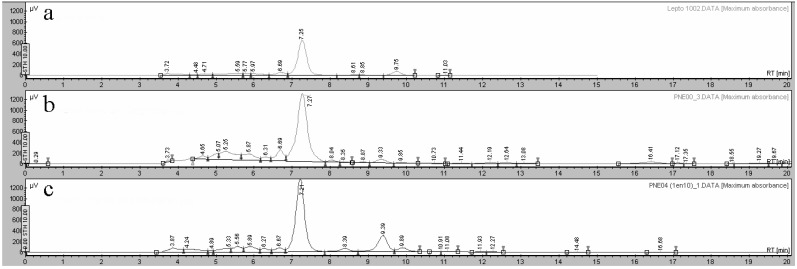
HPLC chromatograms obtained for: (**a**) LTC pure compound; (**b**) extract of *L. rivularis* plants collected from natural habitat; (**c**) extract of micropropagated explants of *L. rivularis.*

The chemical composition of extracts of *L. rivularis* has been extensively studied and it is well established that they contain sesquiterpene lactones, of which LTC is the major component [5,6,30,31]. On the other hand, as the phytochemical composition of plants propagated under in vitro conditions could vary due to controlled cultivation conditions, it becomes necessary to determine the composition of extracts obtained from propagated explants and callus generated in vitro. Thus, the phytochemical composition of extracts obtained from plants collected from their natural habitat, callus, and clonal explants was characterized by HPLC and using a pure sample of LTC as standard. The comparison of the obtained chromatographic profiles is shown in Figure 3.

Interestingly, similar phytochemical compositions were observed for extracts obtained from plants and micropropagated explants, and LTC was shown to be the major component in both extracts. In addition, LTC was not detected in callus extract.

### 2.4. Asseessment of Biological Activity of In Vitro Plant Extracts

In previous work, it was shown that both LTC and extracts of *L. rivularis* exhibit antiproliferative activity against different cancer cell lines [5,6,8,10]. In this work, the cytotoxicity of extracts, obtained from in vitro-propagated plants of *L. rivularis*, was evaluated by using cancer and normal cell lines, namely, HeLa (cervical adenocarcinoma) and CCD841/CoN (colon epithelium non-cancer cell line), respectively. Cell viability was evaluated using the Resazurin colorimetric assay, which is an indirect method that measures cellular metabolic capacity variations. As result of cell metabolism, resazurin is reduced by oxide-reductases found in the mitochondria of viable cells and, consequently, a color change or fluorescence emission can be detected [32,33]. For cells growing under normal conditions, the absorbance can be taken as proportional to cell density and therefore a decrease in absorbance implies a decrease in cell viability.

In this work, changes in resazurin absorbance were determined for untreated cells (T0), cells treated with LTC (T1), *L. rivularis* extract (T2), and DMSO at lethal concentration (positive control).

**Figure 4:**
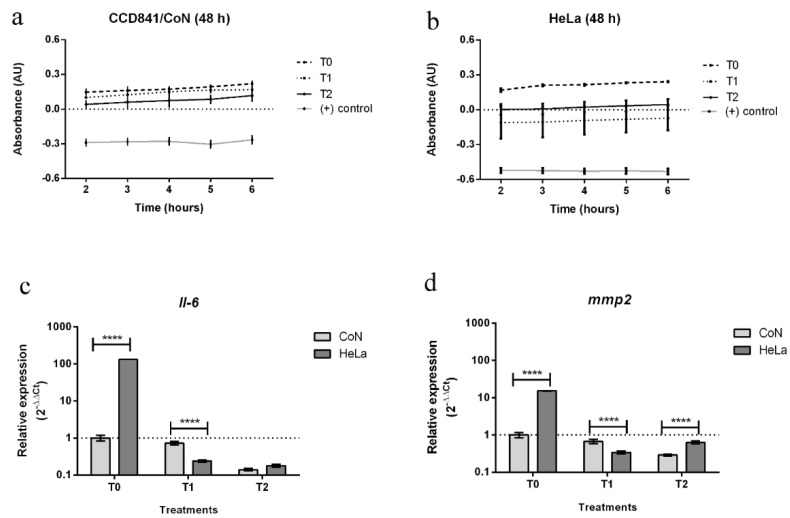
Biological activity of in vitro plant extracts. Resazurin colorimetric assay of: (**a**) control cell line, CoN; (**b**) HeLa cancer cell line. Relative fold change expression: (**c**) IL-6, (**d**) mmp2. All data represent the means of three independent replicates ± SD (*n* = 9). The significance of difference was analyzed by Sidak’s multiple comparisons test (*p* < 0.05); asterisks indicate significant differences compared with negative control under the same treatment conditions (**** *p* < 0.0001).

Negative changes in absorbance were obtained for both of the cell lines submitted to treatment with a high concentration of DMSO (see Figure 4a,b). These negative values indicate that all metabolic process were stopped due to cell death.

On the other hand, cells treated with LTC (1.2 ppm) showed a statistically significant decrease in cell metabolism as compared with untreated cells T0 (0.1% DMSO). Interestingly, the decrease in cell viability after 48 h of exposure to LTC treatment was higher for HeLa than CoN cells. For cancer cells, negative absorbance values were measured, suggesting that a lethal level of LTC was reached, whereas for CoN cells and at the same LTC concentration only a decrease in their metabolic activity was observed. This result is in line with previous studies that have shown that LTC acts specifically on tumor cells of different origin [8,10,11]. Finally, treatment with the extract of clonal explants (T2, 12 ppm) induced a decrease in metabolism in both cell lines but without reaching lethal levels. The data in Figure 4b suggest that a lethal level could be reached at a higher extract concentration. It is worth mentioning that T1 and T2 concentrations were chosen based on the IC_50_ values previously reported for different cancer lines [6,10], and therefore the concentration producing the highest antiproliferative effect can still be optimized. However, these results confirm that LTC is the most active metabolite in *L. rivularis* extract, and that clonal plant extracts could be considered as potential antiproliferative agents.

To delve into possible LTC mechanisms of action, we have analyzed the expression of *Il-6* and *mmp2* genes, which encode interleukine-6 and matrix metalloproteinase 2, respectively. It is well established that IL-6 regulates immune response and cancer cell proliferation [34,35,36,37], whereas MMP-2 promotes cell metastasis processes [38].

In Figure 4c,d is plotted, in a logarithmic scale, the relative fold change expression of *Il-6* and *mmp2* in CoN and HeLa cells after 48 h of treatment with LTC (T1) and the extract of clonal explants (T2). Measurements were performed at 48 h because gene expression reaches a peak after this cell culture period. Our results show that, in the absence of any treatment (T0, 0.1% DMSO), the expression levels of both genes are higher in cancer cells than in normal cells, which is in line with what has been previously reported [39]. Interestingly, LTC (T1) induced a drastic decrease in the expression of both genes in HeLa cells, i.e., the ratio of relative expression, T0/T1, is 720 and 45 for *Il-6* and *mmp2*, respectively. However, LTC had a much lower effect on CoN cells, i.e., T0/T1 values were 1.4 and 1.5 for *Il-6* and *mmp2* expression, respectively. In other words, these results confirm that LTC effects are somehow more heavily expressed on cancer cell lines than in normal cells. Even though there are no studies of the effect of LTC on the expression of these two marker genes, it has been reported that the antitumor activity of natural compounds and extracts is related to the decreasing expression of genes encoding IL-6 and MMP-2 [40,41,42,43]. It has been shown that the inhibition of IL-6 occurs by the interruption of the autocrine signal dependent on the IL-6/STAT3 system. STAT3 and NF-κβ are transcription factors that are activated and maintain a signaling crosstalk in the tumor environment. Both mechanisms induce tumor cell proliferation, survival, invasion, and chemo resistance [44,45,46]. On the other hand, the LTC-induced decrease in *mmp2* expression implies that LTC should reduce metastasis in addition to antiproliferative and apoptosis effects, attributed to the inhibition of the NF-κβ signaling pathway [10].

Finally, the data shown in Figure 4c,d indicate that T2 reduces gene expression in cancer cells in a similar way. The ratio of the relative fold change expression of *Il-6* and *mmp2* (T0/T2) is approximately 720 and 24 for HeLa cells, and 7 and 3.5 for HeLa and CoN cells, respectively. However, the most striking result is the strong effect of T2 on normal cells, which takes the ratio T0/T2 values far from the unity observed for LTC. In other words, it turns out that T2 affects normal cells in a way that LTC does not. This difference could be attributed to other active compounds that are present in the extract at lower concentrations. Overall, this tendency indicates a greater effect of T2 on gene expression in HeLa than in CoN cells, but it is worth mentioning that any decrease in the expression of these genes in cancer or normal cell lines should help to prevent or avoid cancer proliferation and metastasis. Thus, we think that clonal extract of *L. rivularis* is a promising alternative for developing anticancer agents.

## 3. Materials and Methods

### 3.1. In Vitro Micropropagation Platform for Leptocarpha Rivularis

Plant material was donated by a private nursery, located in O’Higgins region, Chile. Non-lignified cuttings measuring 0.3 cm wide by 10 cm in length were used for in vitro culture. Introduction of plant material was carried out using a standard disinfection protocol; briefly, plant material was washed for 20 min with commercial soap and one hour with commercial antifungal agents in doses recommended by the manufacturers, and sterilized for 10 min using a 10% sodium hypochlorite solution. Then, cuttings were sectioned into 2 cm fragments, trying to conserve two axillary buds in each, and transferred to semi-solid medium, which was formulated in this research. To induce axillary buds to sprout, the introduction medium was used for 20–30 days. The medium was used for the propagation and elongation of explants. This culture medium must be renewed every 30–45 days. The extension for over 60 days in this medium generates the rooting of explants. All micropropagation stages were carried out at constant temperature, 24 ± 2 °C, relative humidity, ranging from 30 to 50%, with photosynthetic photon flux density (PPFD) at 40 μmol m^−2^ s^−1^, provided by cool white fluorescent tube lights (Philips, Kolkata, India), and a photoperiod of 16/8 h light/dark.

### 3.2. Callus Induction of Leptocarpha Rivularis

The callogenesis process was tested using various combinations of phytohormones considering several concentrations and information referring to callogenesis processes in the genus *Asteraceae* (see Table 2), observing the effect of these on callus induction of material from lateral leaves and internodal segments obtained from plantlets multiplied in vitro. The induction conditions used considered periods of darkness for 30 days and subsequently, a photoperiod regime of 16 h light/8 h darkness (16/8), or an immediate period of 16/8, without going through the 30 days in darkness. In addition, variables such as temperature (23 ± 2 °C) and humidity (50–70% RH) remained constant. According to the structure’s evolution, media renovation proceeded every 2 to 3 months. Callus formation response was determined according to callus induction index (CIF) [47]. Variables were volume (wet weight/dry weight), callification time, morphology (friable, compact, oxidized), and the presence or absence of organogenesis or other structures [25] (Table 2). Histological sections were performed using widely described protocols [48]. Structures fixed in a FAA solution were embedded in paraffin and subsequently, cuts between 8 to 10 µm were made using microtome (Microm HM325, ThermoFisher, Waltham, MA, USA). These were stained with Safranin-O according to published protocols [49].

### 3.3. Extraction and Composition Analysis of Leptocarpha Rivularis Extracts

Extracts of *L. rivularis* were obtained from plant material generated under in vitro conditions, i.e., total plant and callus extracts. Extract obtained from plants collected from their natural ecological niche was provided by Dr. Héctor Carrasco. Briefly, clonal plantlets without rooting and callus originated from internodal segments were dehydrated at room temperature for 5 days (plants) or by lyophilization for 48 h (callus) in a freeze dryer (Ílshin Biobase, Dongducheon-si, Korea). Dried material was macerated in ethyl acetate (EtAc) for two days, and the solvent was removed under reduced pressure at 50 °C using a rotary evaporator (Buchi, Essen, Germany). Phytochemical profiles of all extracts were determined by high-performance chromatography (HPLC). Chromatograms were obtained with an HPLC instrument (Jasco, Tokyo, Japan) formed by a quaternary pump (PU-2089, Jasco) coupled with a high-sensitivity diode array detector (MD-2010, Jasco), covering from 195 nm to 650 nm with 1 nm of minimum wavelength interval. Semi-polar extract samples (20 uL) were injected onto a C18 column (Kromasill, KR100-5-C18 4.6 × 150 mm). Acetonitrile (ACN):water mixtures of increasing polarity (80%, 70%, 60%) were used as mobile phase at a constant flow of 0.5 mL/min (50 MPa max.). Pure LTC, isolated and characterized in previous work, was used as standard.

### 3.4. Biological Activity of the Total Extracts of Leptocarpha Rivularis In Vitro

Biological activity of LTC was analyzed by means of a cell viability assay and quantification of expression of genes associated with cellular processes of cell proliferation. HeLa ATTCC^®^ CCL2™ (cervical adenocarcinoma) and CCD841/CoN (colon epithelium non-cancer cell line) cell lines were used. Tests were performed under standard culture conditions, and Dulbecco’s modified Eagle’s medium (DMEM, Gibco, Waltham, MA, USA) with no added antibiotics and supplemented with 10% inactive fetal bovine serum was used as basal medium under controlled conditions of CO_2_ (5%) and temperature (37 °C) optimal for both cell lines. For cell viability assay, Resazurin colorimetric assay (Sigma-Aldrich, St. Louis, MO, USA) was used according to the supplier’s recommendations. This assay was performed as follows: in 96-well plates, 10^3^ cells/well were seeded with 200 uL of basal medium for 24 h. Subsequently, basal medium was replaced with basal medium containing LTC (T1, 1.2 ppm) or total extract from in vitro plant material (T2, 12 ppm) [6], which were dissolved in 0.1% DMSO. Negative control was wells containing culture medium with the same volume of 0.1% DMSO added (T0). DMSO at higher concentration (10%) was used as the positive control to induce cell death, because it has been shown that doses greater than 1% induce cell death [50]. Finally, 100 uL of resazurin was added to each well, and maintained under cell culture conditions for an incubation period of 2 h. Color change, induced by a reduction in resazurin, was quantified by measuring absorbance at 530 nm with an Infinite 200 PRO (TECAN, Municipality, Switzerland) at 2, 3, 4, 5, and 6 h after incubation. This assay was carried out at 24, 48 and 72 h after treatment application and each well was measured in triplicate.

For gene expression assays, 10^5^ cells/mL were seeded in 35 mm plates (p6) using cell culture media and under the same culture conditions as those previously described. The material was collected after 48 h of exposure to different treatments using cell lysis solution from Total RNA extraction kit (Macherey-Nagel, Düren, Germany) and extraction was carried out according to the supplier’s instructions. RNA amount was determined using an Infinite 200 PRO spectrophotometer. Gene expression analysis was performed using quantitative real-time polymerase chain reaction (qRT-PCR) using Light Cycler 96 equipment (Roche, Basel, Switzerland). RNA transcription and amplification was completed using Takyon™ One-Step MasterMix (Eurogentec, Seraing, Belgium). Reactions were carried out in triplicate, using previously described primers for carcinogenic marker genes, metalloproteinase-2 enzyme (*mmp2*) and interleukin-6 cytokine (*Il-6*), together with hypoxanthine phosphoribosyltransferase-1 enzyme (*hprt-1*), corresponding to the endogenous gene of constitutive expression [51,52,53]. Ct values for each treatment were normalized by the expression of the *hprt-1* gene, and quantified according to the 2^−ΔΔCt^ methodology [54]. Statistical analyses for the viability test and to determine the changes in gene expression were performed using GraphPad Prism 6 software with a two-way ANOVA and subsequently, mean values with less significant differences of *p* < 0.05 were considered according to Sidak’s multiple comparisons test.

## 4. Conclusions

The clonal micropropagation of *L. rivularis* and callus induction from the internodal segment of in vitro-generated explants were successfully accomplished. The chemical composition of in vitro plant extracts and callus was determined by HPLC and compared with extracts obtained from adult plants grown in natural habitats. Interestingly, the results indicate that both kind of extracts have a similar chemical composition, and that LTC is not present in callus extracts. On the other hand, the cell viability and gene expression of *Il-6* and *mmp2*, genes associated with carcinogenic activity, were measured for HeLa and CoN cells, in the presence and absence of LTC and the extract of micropropagated *L. rivularis* plants. The results show that LTC and the extract exhibit comparative antiproliferative activity, i.e., the diminution of cell viability is more important in cancer cells than in normal cells. Similar behavior was observed for the decrease in gene expression. This effect has not been previously evidenced for *L. rivularis* and suggests the existence of an alternative mechanism of action for their extract and LTC, namely one that directly affects NF-κβ activity and the regulation of the cell cycle, and another affecting the activity of metalloproteinases, and therefore inhibiting the metastasis process of cancer cells.

## Figures and Tables

**Table 1:** Medium composition for in vitro clonal micropropagation platform of *Leptocarpha rivularis*. Murashige and Skoog medium, MS, with 3% (*w/v*) of sucrose, ascorbic acid (0.05 g/L), charcoal (1 g/L) and different hormone combinations. Hormones included are: 1-naphthaleneacetic acid (NAA), 6-benzylaminopurine (BAP), indole-3-butyric acid (IBA) and gibberellic acid (GA_3_). * Indicates best medium protocol.

		Introduction (I)					Propagation (P)			Rooting (R)
		I1	I2	I3	I4	I5 *	P1 *	P2	P3	R1
Hormones (µM)	BAP	2.20	0.44	0.44	3.50	4.40	4.40	4.40	4.40	3.50
	IBA		0.49	0.49	0.49	0.49	0.49		0.49	0.49
	NAA							0.27		
	GA3			0.14	0.14	0.14		0.14	0.14	

**Table 2:** Medium composition for *Leptocarpha rivularis* callus induction. Different combinations of auxins and cytokines were used: 1-naphthaleneacetic acid (NAA), 6-benzylaminopurine (BAP), isopentenyl adenine (2ip), kinetin (KIN), thidiazuron (TDZ), zeatin (Z). n.c.i.: no callus induction.

		Hormones						
Induction Media	Ref	Auxins	Conc. (µM)	Cytokines	Conc. (µM)	CIF (%) * (Mean ± S.E.)	Plant Organ	Callus Charact ^†^
C0						0	leaf, intermodal section	n.c.i.
C1		NAA	2.7	TDZ	4.5	0	leaf	n.c.i.
C2		NAA	2.7	KIN	4.6	0	leaf	n.c.i.
C3		NAA	2.7	KIN	2.3	33.3 ± 1.2	leaf	compact, rooting
C4		NAA	5.5	KIN	2.3	0	leaf	n.c.i.
C5		NAA	5.5	KIN	2.3	0	leaf	n.c.i.
C6	[24]	NAA	5.5	BAP	4.4	0	root	n.c.i.
C6	[24]	NAA	5.5	BAP	4.4	36.1 ± 2.6	leaf	compact, greenish
C7		NAA	2.7	BAP	4.4	94.4 ± 1.5	internodal section	friable, green
C8		NAA	5.5	BAP	4.4	91.8 ± 1.3	internodal section	friable, green
C9	[21]	2,4D	4.9	BAP	4.4	0	leaf	n.c.i.
C10	[21]	2,4D	9.8	BAP	8.8	27.8 ± 3.2	leaf, root	compact, brown
C11	[21]	2,4D	13.5	BAP	8.8	0	leaf	n.c.i.
C12				BAP-KIN		0	leaf, intermodal section	n.c.i.
C13				BAP-Z		2.7 ± 0.6	leaf	compact, rooting
C14				2ip		0	leaf	n.c.i.

* CIF (%) = number of explants forming calluses/(total number of explants × 100%). ^†^ Callus description following Ikeuchi et al. 2013 [25].

## Data Availability

Not applicable.
